# Predictors of microvascular invasion in patients with solitary small hepatitis B related hepatocellular carcinoma

**DOI:** 10.12669/pjms.302.4652

**Published:** 2014

**Authors:** Zhen You, Li-Ping Chen, Hui Ye

**Affiliations:** 1 Zhen You, Division of Biliary Surgery, West China Hospital of Sichuan University, Chengdu (610041), China.; 2 Li-Ping Chen, Division of Biliary Surgery, West China Hospital of Sichuan University, Chengdu (610041), China.; 3 Hui Ye, Division of Biliary Surgery, West China Hospital of Sichuan University, Chengdu (610041), China.

**Keywords:** Hepatocellular carcinoma, Microvascular invasion, Predictor

## Abstract

***Objective: ***To identify the risk factors for the presence of microvascular invasion (MVI) in patients with solitary small hepatitis B related hepatocellular carcinoma (HCC).

***Methods: ***The data of 215 patients who underwent liver resection between 2008 and 2011 at our hospital were reviewed. MVI was confirmed on pathological examination in 41 patients. Preoperative risk factors for MVI were analyzed using uni- and multi-variate analyses.

***Results:*** In the multivariate analysis, alpha-fetoprotein (AFP) greater than 400 ng/mL, tumor size and hypersplenism were independently associated with MVI. Receiver operating curve (ROC) analysis suggested the best cut-off value for tumor size was greater than 3.1 cm. The ROC curve analysis further identified patients with more than one above-mentioned risk factor may suffer from MVI with 75.6% sensitivity and 75.3% specificity. The recurrence-free and long-term survival rates of patients with MVI were significantly lower than patients without MVI.

***Conclusions:*** Patients with MVI may suffer from poor outcomes. AFP greater than 400 ng/mL, tumor size and hypersplenism were preoperative predictors of MVI in patients with solitary small hepatitis B related HCC.

## INTRODUCTION

Hepatocellular carcinoma is the sixth most common cancer and third most frequent cancer-related death in the worldwide.^1^ Owning to the highly prevalence of hepatitis B infection, the incidence of HCC in China alone accounts for about 55% cases in the worldwide.^2^ Liver resection is perceived as a curative treatment for HCC in cirrhotic patients with good functional liver reserves. However, many risk factors may contribute to the postoperative recurrence after curative liver resection, such as tumor size, tumor numbers, high preoperative AFP level and so forth.^3^^,^^4^ Microvascular invasion (MVI) is a strong prognostic factor for HCC after liver resection, even after liver transplantation. A number of investigations suggested MVI impact significantly on long-term and recurrence-free survivals after liver resection and transplantation.^3^^,^^5^ The cancer cells may have spread via the vasculature before liver resection or transplantation for HCC in patients with MVI.^6^ Macrovascular invasion can be detected by preoperative imaging, however, different to macrovascular invasion, detection of MVI using preoperative radiological imaging still remains a difficulty.^7^^,^^8^ Histological examination is still the only accurate method for assessing MVI until now.^9^ Although previous investigations indicated tumor size may be a predictive factor for MVI, which contributes to MVI for patients with small HCC is still unclear. Accordingly, we retrospectively reviewed our data to evaluate the possible risk factors for MVI in patients with small HCC.

## METHODS

***Study group: ***The data of patients with single small HCC (no more than 5 cm in diameter) who underwent liver resection from 2008 to 2011 at our hospital were reviewed. HCC and microvascular invasion were confirmed by pathological examination after liver resection. According to the presence of MVI, patients were divided into two groups. Group MVI(+), which consisted of patients with MVI; group MVI(-), which consisted of patients without MVI. Hypersplenism was defined as follow: platelet counts less than 80 ×10^9^/L and/or white blood cell counts less than 3×10^9^/L.^10^ This study was approved by the ethics committee of West China Hospital.

***Surgical procedure: ***Patients who received liver resection at our hospital should have preoperative liver function of Child-Pugh class A. Hemihepatic vascular occlusion was the primary technique performed to reduce intraoperative bleeding. The Pringle manoeuver was considered as a back-up strategy. A CUSA Excel™ device was utilized for liver transection. Drainage was routinely placed in the subphrenic cavity before closure.

***Follow-up: ***After operation, all patients were monitored by serum AFP examination, visceral ultrasonography or CT or MR imaging and chest radiography every three months. Bone scintigraphy was performed whenever HCC recurrence was suspected. Recurrence was defined as positive imaging findings compared to preoperative examination values and newly rising tumor marker (AFP) values or confirmed by biopsy or resection.^11^

***Statistical analysis: ***Continuous variables were presented as the mean±SD. Categorical variables were analyzed using the chi-square test or Fisher’s exact test, whereas one-way analysis of variance was used to analyze continuous variables. Independent risk factors were identified by logistic regression. Factors significant at a *P* < 0.10 in the univariate analyses were involved in the multivariate analyses. The diagnostic accuracy of the identified risk factors was evaluated using receiver operating curve (ROC). The Kaplan-Meier method with log-rank test was utilized to compare the long-term survival of the two groups. A *p *value of less than 0.05 was considered as statistically significant.

## RESULTS

***Demographic data of current study: ***A total of 215 patients were included in the present study, including 182 males and 33 females. In the present study, MVI was observed in 41 patients. The mean age was 50.31±10.98 years. The mean tumor size was 3.33±1.05 cm. In current study, the preoperative AFP level of 74 patients was higher than 400 ng/mL. Hypersplenism was observed in 70 patients. The HBV-DNA level of 114 patients was positive in the present study.

***Risk factors of microvascular invasion: ***In the univariate analyses, tumor size, positive HBV-DNA level, preoperative AFP level greater than 400 ng/mL and hypersplenism were potential risk factors for MVI (Table-I). However, in the multivariate study, only tumor size, preoperative AFP level greater than 400 ng/mL and hypersplenism were independent predictors for MVI (Table-II). The ROC curve analysis showed that the best cut-off value of tumor size for identifying MVI was greater than 3.1 cm with 92.93% sensitivity and 52.3% specificity. The area under the ROC curve was 0.716.

A simple preoperative prognostic score was derived from the results of the multivariate analysis. Each above-mentioned risk factor was given a score of 1. If patient had all of the above-mentioned risk factors, the prognostic score was 3; if patient had any two of the 3 independent risk factors, the prognostic score was 2; if patient had any one of the 3 independent risk factors, the prognostic score was 1; if patient didn’t have any risk factor, the prognostic score was 0. The ROC curve analysis showed the best cut-off value for the prognostic score was greater than 1 risk factor with 75.6% sensitivity and 75.3% specificity. The area under the ROC curve was 0.790.

**Fig.1:**
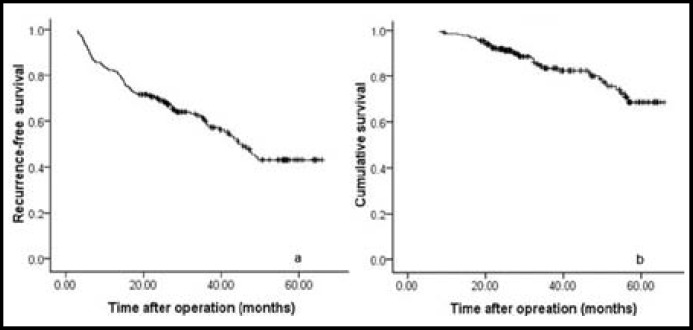
The recurrence-free (a) and long-term (b) survival curves of all patients

**Fig.2:**
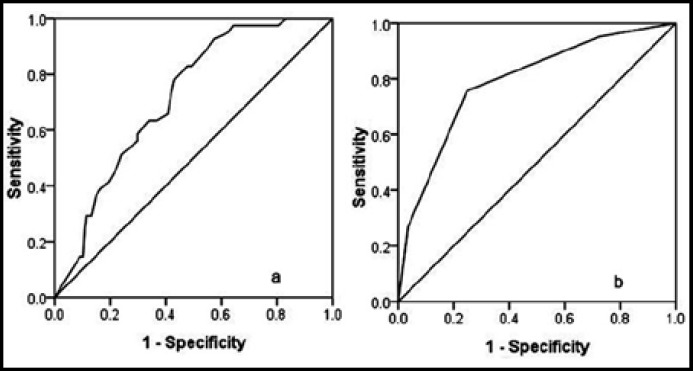
Receiver operating curves for the tumor size (a) and the predictive risk factors (b) that were confirmed by multivariate analysis

***Long-term survival comparison of patients with and without MVI: ***In the present study, recurrence was observed in 95 patients. Of the 95 patients with postoperative recurrence, 38 patients died. The overall 1-, 3-, 5-year recurrence-free survival rates for all patients were 81.9%, 59.9% and 43% respectively (Fig.1a); whereas the overall 1-, 3-, 5-year long-term survival rates for all patients were 98.1%, 83.5% and 68.6% respectively (Fig.1b). The 1-, 3-, 5-year recurrence-free survival rates of group MVI(-) were 82.8%, 62.8% and 48.5% respectively, which were significantly higher than the group MVI(+) (78%, 44.9% and 24.7 respectively; Fig.2a; *P*=0.025). The 1-, 3-, 5-year long-term survival rates of group MVI(-) were also significantly higher than the group MVI(-) (98.3%, 89.2%, 74.6% versus 80.1%, 46.2%, 46.2%; Fig.2b; *P* < 0.001).

## DISCUSSION

MVI is widely accepted as a strong prognostic factor for poor outcomes after liver resection and liver transplantation. For patients with MVI, cancer cells may have disseminated to intrahepatic and/or exhepatic sites via the vascular culture vasculature before liver resection or liver transplantation. Recently, Lim et al.^9^ confirmed MVI is a better predictor to Milan criteria in prediction postoperative recurrence after surgical resection for HCC. Their investigation also suggested patients with MVI may suffer from a higher incidence of early recurrence than patients without MVI.^9^ In our current study, we also confirmed patients with MVI had an increased risk of postoperative recurrence and reduced long-term survival rate.

**Table-I:** Univariate analyses of risk factor for MVI

*Variables *	*MVI(-) N=174*	*MVI(+) N=41*	*P*
Gender (female)	28	5	0.636
Age (years)	49.79±10.82	52.54±11.50	0.149
Tumor size (cm)	3.20±1.04	3.98±0.79	< 0.001
Differentiation			0.796
Well	17	4	
Moderate	88	23	
Poor	69	14	
HBV-DNA (+)	87	27	0.082
AFP > 400ng/mL	50	24	< 0.001
Total bilirubin (μmol/L)	16.01±6.63	15.50±6.47	0.657
INR	1.06±0.11	1.03±0.12	0.105
Albumin (g/L)	40.93±6.33	42.15±4.59	0.343
Platelet (10^9^/L)	107.36±48.25	114.54±56.95	0.410
AST (U/L)	24.77±17.67	22.15±7.26	0.353
ALT (U/L)	26.75±20.78	25.37±15.47	0.690
Prothrombin time (s)	12.00±1.34	11.77±1.50	0.343
Hypersplenism	47	23	0.001

**Table-II:** Multivariate analyses of risk factor for MVI

*Variables *	*Coefficient*	*OR*	*95% CI*	*P*
Tumor size (cm)	0.914	2.493	1.625-3.825	< 0.001
AFP > 400ng/mL	1.386	4.000	1.827-8.758	0.001
Hypersplenism	1.320	3.745	1.625-3.825	< 0.001

**Fig.3:**
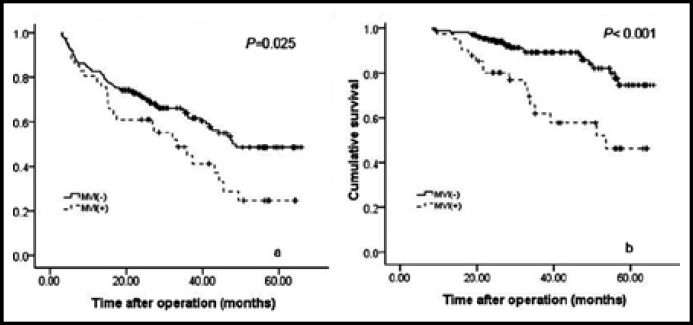
Recurrence-free (a) and long-term (b) survival comparison of patients with and without MVI.

Preoperative AFP level was an independent risk factor associated with MVI in the present study. Previous investigations suggested AFP may be a marker of tumor aggressiveness.^12^^,^^13^ Experimental studies confirmed inhibition of AFP mRNA expression could inhibit the proliferative activity in HCC cell line.^14^^,^^15^ Recently, Canly et al.^16^ suggested AFP-specific immunotherapy could also inhibit the growth of autochthonous hepatocellular carcinoma in mice. This could also explain the correlation of higher AFP level and MVI. AFP was even used to stage HCC or used to select liver transplant candidates.^17^ Moreover, a number of investigations have also confirmed higher preoperative AFP level was associated with higher recurrence rate and poor outcomes for patients with HCC.^3^^,^^18^ Graham et al.^19^ even suggested liver transplantation should offer to patients with small solitary HCC and high preoperative AFP level rather than liver resection.

Consistent with previous studies, our study also confirmed the association between tumor size and MVI.^20^ Esnaola et al.^21^ suggested tumor size greater than 4 cm was a risk factor for MVI in patients with HCC within Milan criteria who were candidates for liver transplantation. Nagano et al.^20^ reported tumor sized greater than 7 cm was associated with MVI. Pawlik et al.^22^ suggested for patients with solitary HCC, tumor size greater than 5 cm significantly predicted MVI. In the present study, the best cut-off value for tumor size was greater than 3.1 cm, which was much smaller than the previous studies. In our study, we only concluded patients with solitary small HCC, whereas large HCC tumors were included in many previous studies. Moreover, the definition of MVI still remains controversial, because some investigators include wall, muscle vessel, or contiguity with the liver parenchyma, whereas others define microvascular invasion as lesions visible only on microscopic examination.^23^^,^^24^ These reason may explain why our results was much smaller than the previous investigations.

Hypersplenism was another independent risk factor for MVI in our study. A lot of investigations suggested liver resection should not be performed to patients with HCC and hypersplenism because of the high incidence of postoperative recurrence.^25^ Our study further suggested patients with hypersplenism had an increased risk of MVI. This result could explain why patients with hypersplenism had a worse outcome than patients without hypersplenism. However, the detail mechanism of the correlation between MVI and hypersplenism is still unclear and needs a further study.

In conclusion, the presence of MVI increased the incidence of postoperative recurrence and reduced the long-term survival rate of patients with HCC. Tumor size, high AFP level and hypersplenism were independent risk factors of MVI for patients with solitary small HCC.
